# MiR-335 promotes corneal neovascularization by Targeting EGFR

**DOI:** 10.1186/s12886-022-02481-0

**Published:** 2022-06-15

**Authors:** Jingjing Qian, Junbo Yu, Xi Zhu, Shu Liang

**Affiliations:** 1 Department of Ophthalmology, Affiliated Hospital of Nantong University, No. 20 Xisi Road, Nantong, 226001 Jiangsu Province China; 2 Department of Trauma Center, Affiliated Hospital of Nantong University, No. 20 Xisi Road, Nantong, 226001 Jiangsu Province China

**Keywords:** Corneal neovascularization, Angiogenesis, Human umbilical vein endothelial cells, miR-335, EGFR

## Abstract

**Background:**

Corneal neovascularization (CRNV) is a severe threat to the vision of people. MicroRNA-335 (miR-335) has the function of facilitating angiogenesis. However, whether miR-335 regulates the progression of CRNV remains unclear.

**Methods:**

The miR-335 expressions in CRNV rats induced by corneal suture and HUVECs induced by b-FGF were detected by quantitative real-time PCR. For the miR-335 function, wound healing and tube formation assays were performed. For the miR-335 mechanism, a dual-luciferase reporter gene assay was conducted. Besides, for the epidermal growth factor receptor (EGFR) function, Cell Counting Kit-8 and wound healing assays were performed. Meanwhile, the rescue assay was used to assess the miR-335/EGFR function in the migration and angiogenesis of b-FGF-treated HUVECs.

**Results:**

Functionally, the miR-335 knockdown weakened the migration and angiogenesis of b-FGF-treated HUVECs, while the miR-335 overexpression showed an opposite trend. Mechanistically, miR-335 interacted with EGFR and negatively regulated the expression of EGFR. The rescue assay illustrated that miR-335 regulated the migration and angiogenesis of b-FGF-treated HUVECs through EGFR.

**Conclusions:**

In general, our data confirmed that miR-335 facilitated the process of CRNV by targeting EGFR.

**Supplementary Information:**

The online version contains supplementary material available at 10.1186/s12886-022-02481-0.

## Introduction

The cornea is a transparent and avascular tissue located in front of the iris and pupil. Keeping transparency and avascularity is critical for maintaining the best vision and protecting the eyes from injury [1]. Once the balance of angiogenic and anti-angiogenic factors that maintain corneal transparency is destroyed, the abnormal new vessels invade the corneal stroma in the precorneal structure, thereby inducing corneal neovascularization (CRNV) [2]. CRNV is a threat to vision and is an increasing public health problem, which leads to corneal edema, persistent inflammation, and vision loss [3]. Although there has been some progression in the treatment of CRNV, unfortunately, the therapeutic effect is still lacking. Thus, it is urgent to find effective targets for CRNV treatment.

MicroRNAs (miRNAs) effectively repress gene expression by integrating into RNA-induced silencing complexes to regulate target molecules' stability and translation efficiency [4, 5]. MiRNAs participate in embryonic development, phylogeny, tissue differentiation, and evolution of diseases [6]. Emerging evidence shows that miRNAs are abnormally expressed in CRNV and are interrelated to regulate the occurrence and development of CRNV. For instance, miR-204 expression is decreased in vascularized cornea and represses the process of CRNV by targeting VEGF and VEGF receptor 2 [7]; the elevated miR-1275 and miR-1246 expressions inhibit the angiogenesis of human umbilical vein endothelial cells (HUVECs), providing promising biomarkers for the clinical treatment of CRNV [8].

As an essential member of miRNAs, miR-335 has been proved to have essential regulatory functions in various human diseases, mainly including hepatocellular carcinoma [9], non-small cell lung cancer [10], and acute ischemic stroke [11]. Notably, accumulated evidence suggests that miR-335 exerts essential roles in the regulation of angiogenesis. For instance, Zhang et al. indicated that miR-335 influences the angiogenesis of tumor cells, which might provide a new therapeutic strategy for the tumor treatment [12]; Desjarlais et al. demonstrated that under the pathological condition of neovascularization, miR-335 is abnormally highly expressed and participates in the regulation of angiogenesis [13]. However, it is not clear whether miR-335 is interrelated to the regulation of CRNV.

In the current study, we found that miR-335 was elevated in CRNV rats induced by corneal suture and HUVECs induced by b-FGF, suggesting that miR-335 might be involved in the regulation of CRNV. Based on these findings, we continue to investigate the function and potential molecular mechanism of miR-335 in CRNV by using various molecular techniques, aiming to provide novel potential targets for CRNV treatment.

## Materials and methods

### Construction of CRNV rat model

Twenty-four female Sprague–Dawley rats (180–200 g) were purchased from Shanghai Lab. Animal Research Center (Shanghai, China). Rats were randomly grouped into control, 3 d, 7 d and 14 d. Each group consisted of six rats. The rat model of CRNV was induced by corneal suture. Under general and topical anesthesia by pentobarbital sodium, rats received three intermittent sutures on the surrounding corneal stroma, each of which was more than 120°. The surgery was performed only on the right eye of rats. Photographs were taken under the slit lamp before surgery and on day 3, 7, 11 and 14. The Animal Research Committee of the Affiliated Hospital of Nantong University approved all animal studies.

### Cell culture and different treatments

HUVECs were from AllCells (Shanghai, China). They were put in an RPMI 1640 medium supplemented with 10% fetal bovine serum (FBS, Gibco, Grand Island, USA), 60 μg/ml of endothelial cell growth supplement (ThermoFisher Scientific, WA, USA) and 100 U/ml of penicillin with 100 μg/ml of streptomycin (Gibco). The cells were incubated in an incubator at 37 °C, with 5% CO_2_.

To simulate the in vitro CRNV models, HUVECs were treated with 5, 10 or 20 ng/ml essential fibroblast growth factor (b-FGF) for 24 h.

### Quantitative real-time PCR

TRIzol reagent (Invitrogen, Carlsbad, USA) was applied to extract the total RNA from cornea tissues and HUVECs. A total of 1 μg RNA and PrimeScript™ RT reagent Kit (TaKaRa, Dalian, China) was conducted to synthesize the complementary DNA (cDNA). For the analysis of miRNA, the cDNA was synthesized using Mir-X™ miRNA First-Strand Synthesis Kit (TaKaRa). Then, the real-time PCR was carried out on ABI 7500 Fast System (Applied Biosystems, Waltham, Ma, USA) with FastStart Essential DNA Green Master (Roche Life Science, Indianapolis, USA), referring to the standard procedure of manufacturers. U6 was applied as a control for miR-335, and β-actin was applied as a control for EGFR. The 2^−ΔΔCt^ method was conducted to evaluate the relative expression of miR-335 and EGFR. All primer sequences are presented in Table 1.

**Table 1:** The sequences of primers used in qRT-PCR

Gene name	Primer sequence (5’-3’)
miR-335	Forward: TCAAGAGCAATAACGAAAAATGT
	Reverse: GCTGTCAACGATACGCTACGT
EGFR	Forward: CTACAACCCCACCACGTACC
	Reverse: CGCACTTCTTACACTTGCGG
β-actin	Forward: GGACTTCGAGCAAGAGATGG
	Reverse: AGCACTGTGTTGGCGTACAG
U6	Forward: CGCTTCGGCAGCACATATAC
	Reverse: TTCACGAATTTGCGTGTCAT

### Wound healing experiment

The HUVECs with different treatments were inoculated in 6-well plates and cultured for 24 h. Followed by the culture, sterile pipette tips were applied to produce the scratch in the monolayer perpendicularly across the center of the well. Next, the suspended cells were washed away with PBS. 24 h later, a microscope (Olympus, Japan) was used to observe and capture images.

### Tube formation assay

According to the previously described methods [14], the tube formation assay was performed. In detail, a total of 70 μl Matrigel (Corning, Bedford, MA, USA) was placed on 96-well plates and put in an incubator at 37 °C for 30 min. Subsequently, HUVECs (1 × 10^4^) were inoculated on the surface of the gel and cultured for 16 h. Ultimately, the number of branches and the tube length were assessed by ImageJ software (National Institutes of Health, Bethesda, MD, USA).

### Cell transfection

The miR-335 inhibitor, miR-335 mimic, si-EGFR and corresponding controls were synthesized from RiboBio (Guangzhou, China). The pcDNA-EGFR and pcDNA were provided by GenePharma (Shanghai, China).

Before transfection, HUVECs were inoculated in 100 mm diameter dishes for 24 h. Then, according to the manufacturer's standard procedure, the miR-335 inhibitor, miR-335 mimic, si-EGFR, PCDNA-EGFR and their corresponding controls were transfected into HUVECs using Lipofectamine™2000 (Invitrogen). Each assay was repeated three times independently.

### Dual-luciferase reporter gene assay

Previously, we predicted that there were binding sites between miR-335 and EGFR. Then, a dual-luciferase reporter gene assay was applied to verify whether miR-335 is bound to the 3'-UTR of EGFR. In brief, HUVECs were inoculated in 96-well plates (3 × 10^3^) and cultured for 24 h. Followed by the culture, a luciferase reporter vector that contained the potential miR-335 binding sites of 3'-UTR of EGFR was constructed. Then the above recombinant vector and miR-335 mimic were co-transfected into HUVECs using Lipofectamine 2000 (Invitrogen). 48 h after transfection, the relative luciferase activity was quantified using the Dual-Glo™ Luciferase Assay System (Promega, Wisconsin, USA).

### Western blot

RIPA lysis buffer (Beyotime Biotechnology, Shanghai, China) was applied to extract total proteins from CRNV tissues and HUVECs. The total proteins were quantitated using a BCA™ Protein Assay Kit (Beyotime Biotechnology). After that, a total of 25 µg of protein were separated on 12% SDS/PAGE gels and transferred onto nitrocellulose membranes (Bio-Rad Laboratories, Hercules, USA). Then, the membranes were blocked with a 5% non-fat milk (BD Biosciences, USA) at room temperature for one h and incubated with the primary antibodies, containing anti-EGFR (Abcam, 1: 2000 dilution, ab52894) and anti-actin (Abcam, 1: 500 dilution, ab8227) at 4 °C overnight. Next, the membranes were incubated with the secondary antibody (Abcam, 1: 2000 dilution, ab205718) for 1.5 h at room temperature. Subsequently, ImageJ software (National Institutes of Health) was used to scan and analyze all the specific bands.

### Cell Counting Kit-8 (CCK-8) assay

Referring to the instructions of the reagent manufacturer, the Cell Counting Kit-8 Assay Kit (Abcam, Cambridge, UK) was conducted to analyze the proliferation of HUVECs. In brief, HUVECs were grown in 96-well plates and cultured for 24 h. Subsequently, ten μl CCK-8 solution was added and incubated at 37 °C for 2.5 h. The proliferation of HUVECs was assessed by a microplate reader (ThermoFisher Scientific) at 0, 24, 48 and 72 h.

### Statistical analysis

Two groups were compared by Student’s t-test, and Two-way ANOVA was used when more than two groups were compared. All these statistical analysis were conducted using GraphPad Prism software (GraphPad Software, USA). The data are presented as mean ± standard deviation of three independent assays. *P* < 0.05 was considered statistically significant.

## Results

### MiR-335 is highly expressed in CRNV rats induced by corneal suture, and HUVECs treated with b-FGF

**Fig. 1:**
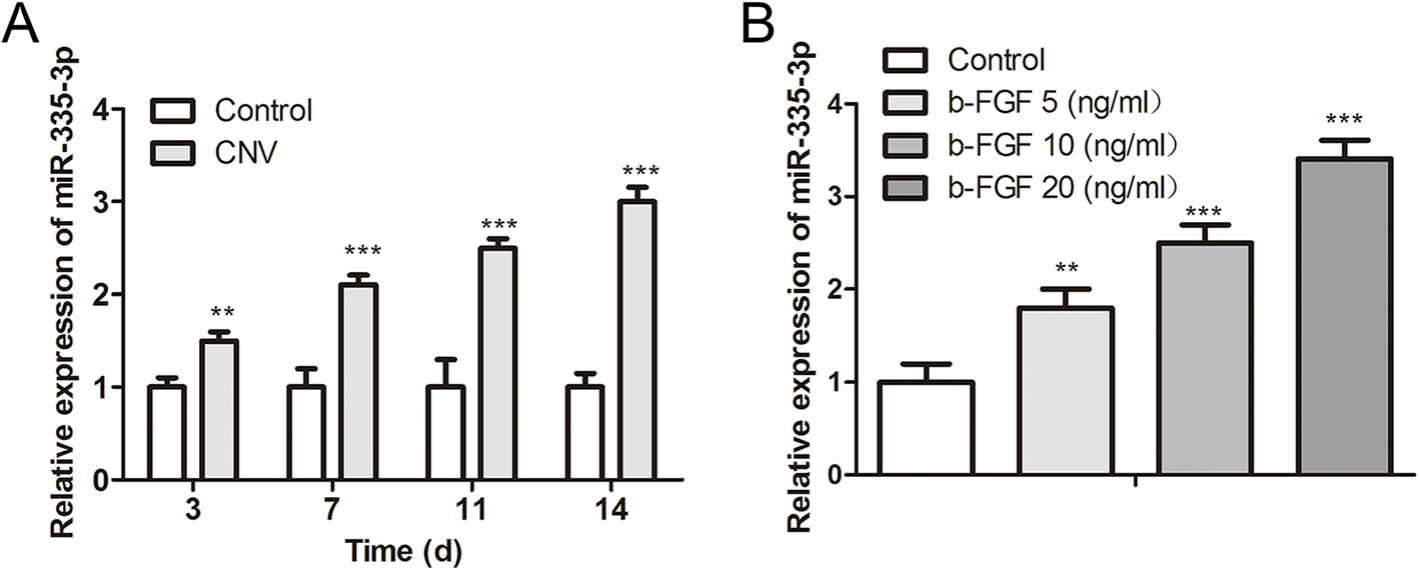
Expression of miR-335 in corneal neovascularization rats induced by corneal suture and human umbilical vein endothelial cells treated with essential fibroblast growth factor. The corneal neovascularization (CRNV) rat model was induced by corneal suture. **A** Quantitative real-time PCR (qRT-PCR) was carried out to measure the miR-335 expression in CRNV tissues of the rats on the day 3, 7, 11 and 14 after surgery. **B** Human umbilical vein endothelial cells (HUVECs) were treated with 5, 10 or 20 ng/ml basic fibroblast growth factor (b-FGF) to establish the in vitro CRNV models. The expression of miR-335 in cells was tested using qRT-PCR. **P < 0.01 ***P < 0.001 vs.Control. CRNV: corneal neovascularization

Firstly, we determined the expression of miR-335 in rat tissues of CRNV. After establishing the CRNV rat model induced by corneal suture, qRT-PCR was carried out to quantify the miR-335 expression on the day 3, 7, 11 and 14 after surgery. The results demonstrated that compared with the control group, miR-335 was increased in the CRNV group, and its expression reached the maximum on day 14 after surgery (Fig. 1A). A previous study indicates that the HUVECs treated with b-FGF can be used to simulate the in vitro CRNV models [15]. As exhibited in Fig. 1B, the miR-335 expression was elevated in the b-FGF group, and the miR-335 expression was elevated with the increased b-FGF concentration. These results confirmed that miR-335 expression was increased in CRNV rats induced by corneal suture and HUVECs treated with b-FGF.

### B-FGF treatment promotes HUVEC migration and angiogenesis

**Fig. 2:**
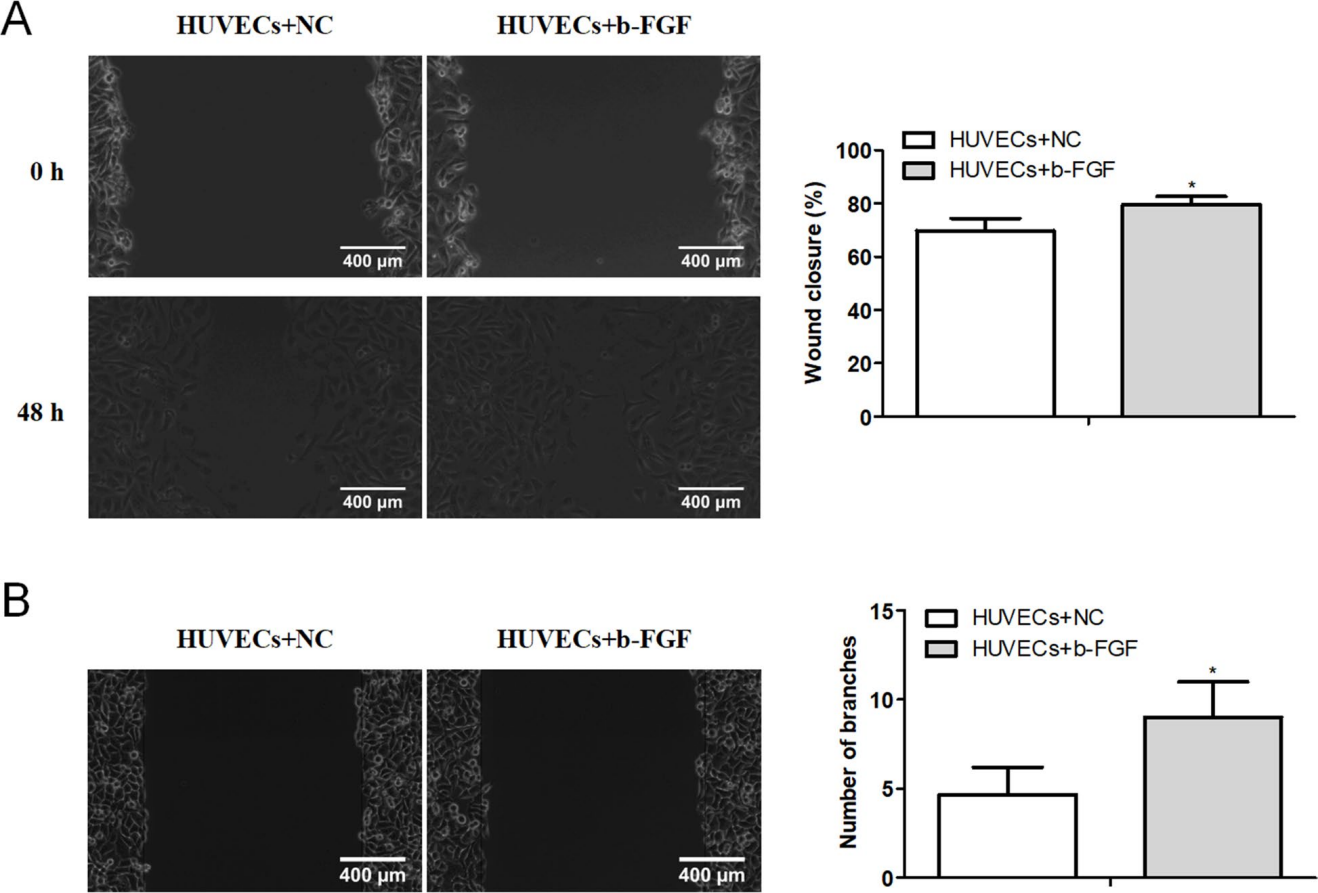
Influence of b-FGF treatment on HUVEC migration and angiogenesis. HUVECs were treated with 20 ng/ml b-FGF for 24 h. **A** A wound-healing assay was applied to assess the migration ability of HUVECs. **B** The number of branches and the tube length was analyzed by tube formation assay (scale bar: 400 μm). *P < 0.05 vs. HUVECs + NC. NC: negative control

Subsequently, we further evaluated the effect of b-FGF treatment on HUVEC migration and angiogenesis. After the HUVECs were treated with b-FGF, the results of wound healing showed that b-FGF treatment enhanced the migration ability of HUVECs (Fig. 2A). Meanwhile, the analysis of tube formation assay illustrated that both the number of branches and the tube length were increased after the b-FGF treatment (Fig. 2B). In general, the above experimental data confirmed that the b-FGF treatment facilitated HUVEC migration and angiogenesis.

### Effect of miR-335 on the migration and angiogenesis of b-FGF-treated HUVECs

**Fig. 3:**
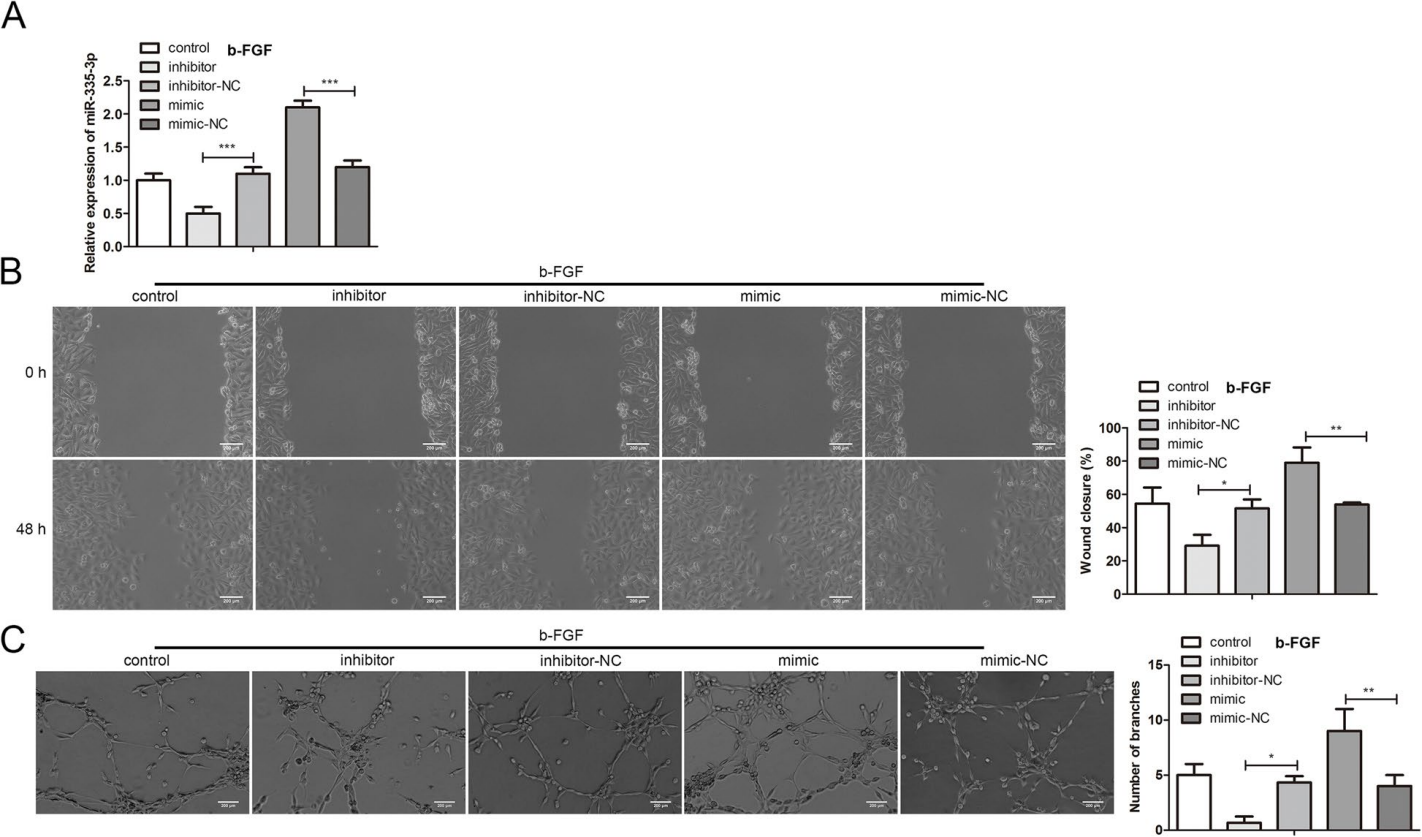
Regulation of miR-335 on the migration and angiogenesis of b-FGF-treated HUVECs. MiR-335 inhibitor or miR-335 mimic was transfected into HUVECs, and then the cells were treated with 20 ng/ml b-FGF for 24 h. **A** qRT-PCR was conducted to verify the transfection efficiency of miR-335 inhibitor and miR-335 mimic. **B** The migration ability of HUVECs was assessed by wound healing analysis. **C** A tube formation assay was applied to analyze the number of branches and the tube length (scale bar: 200 μm). *P < 0.05, **P < 0.01, ***P < 0.001

To clarify the regulation of miR-335 on the migration and angiogenesis of b-FGF-treated HUVECs, miR-335 inhibitor or miR-335 mimic was transfected into HUVECs. Then the cells were treated with b-FGF. As displayed in Fig. 3A, miR-335 was decreased after the transfection of the miR-335 inhibitor. On the contrary, the miR-335 expression was elevated after the transfection of miR-335 mimic, suggesting that we successfully knocked down or overexpressed miR-335 in HUVECs. Based on this, wound healing analysis indicated that the miR-335 knockdown weakened the migration ability of HUVECs, and the miR-335 overexpression produced the opposite trend (Fig. 3B). Moreover, the transfection of miR-335 inhibitor reduced the number of branches and the tube length, and the transfection of miR-335 mimic showed an opposite effect (Fig. 3C). To sum up, the above data indicated that the miR-335 knockdown weakened the migration and angiogenesis of b-FGF-treated HUVECs, and the miR-335 overexpression showed an opposite effect.

### MiR-335 interacts with EGFR

**Fig. 4:**
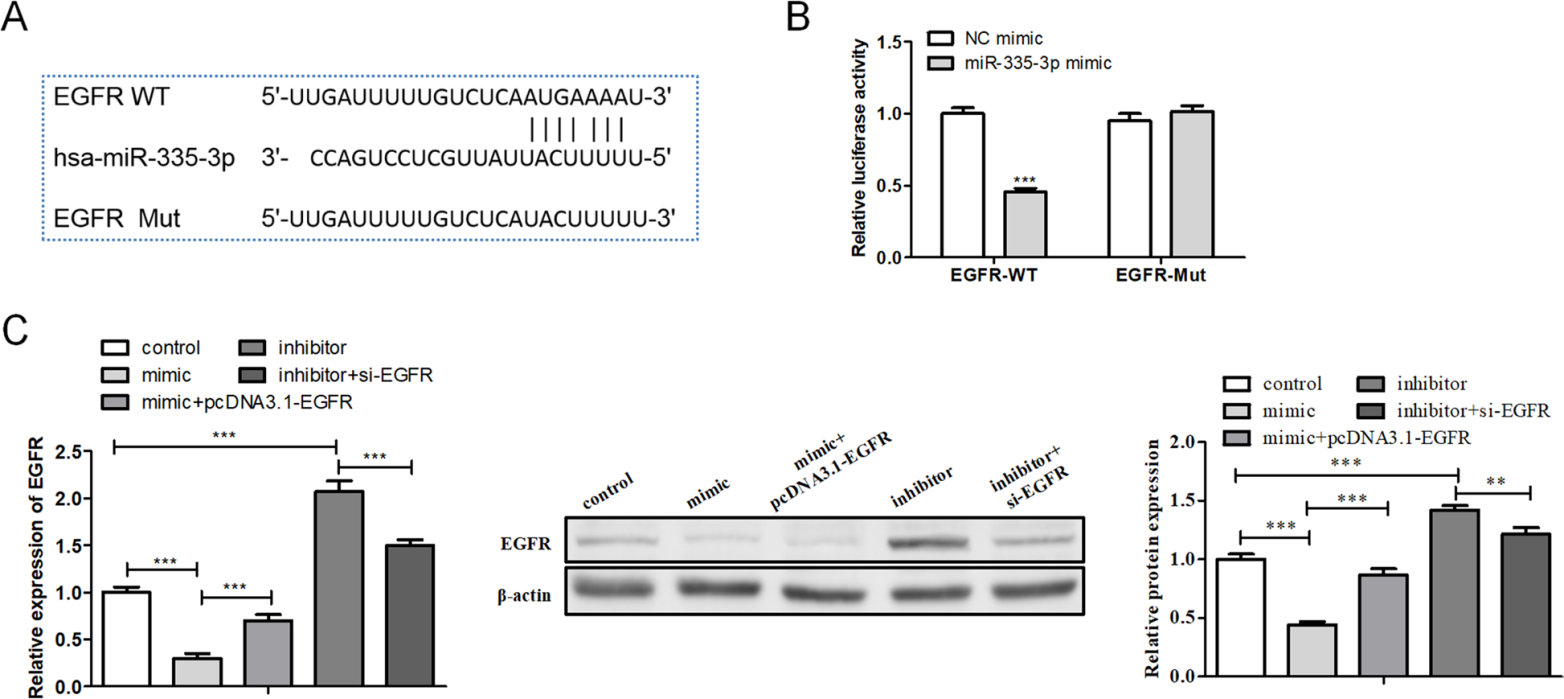
Verification of the interaction between miR-335 and Epidermal Growth Factor Receptor. **A** The binding sites between miR-335 and epidermal growth factor receptor (EGFR) were predicted. **B** A dual-luciferase reporter assay was conducted to confirm the interaction between miR-335 and EGFR. **C** MiR-335 mimic and pcDNA-EGFR, miR-335 inhibitor and si-EGFR into HUVECs. The mRNA and protein levels of EGFR were determined using qRT-PCR and Western blot. **P < 0.01, ***P < 0.001. WT: wild type; Mut: mutant

Previously, we predicted that there were binding sites between miR-335 and EGFR (Fig. 4A). Then, the dual-luciferase reporter assay further revealed that miR-335 negatively regulated the relative luciferase activity of EGFR WT but had no apparent changes in the EGFR MUT relative luciferase activity, which confirmed that miR-335 interacted with EGFR (Fig. 4B). Furthermore, we evaluated the regulation of miR-335 on EGFR expression by transfecting miR-335 mimic and pcDNA-EGFR, miR-335 inhibitor and si-EGFR into HUVECs. As shown in Fig. 4C, the transfection of miR-335 mimic down-regulated the mRNA and protein levels of EGFR, and this down-regulation was partially reversed after the overexpression of EGFR; the transfection of miR-335 inhibitor up-regulated the EGFR mRNA and protein levels, and this up-regulation was partially reversed after the transfection of si-EGFR. In summary, miR-335 bound to EGFR and negatively regulated the expression of EGFR.

### EGFR is lowly expressed in CRNV rats induced by corneal suture, and HUVECs treated with b-FGF

**Fig. 5:**
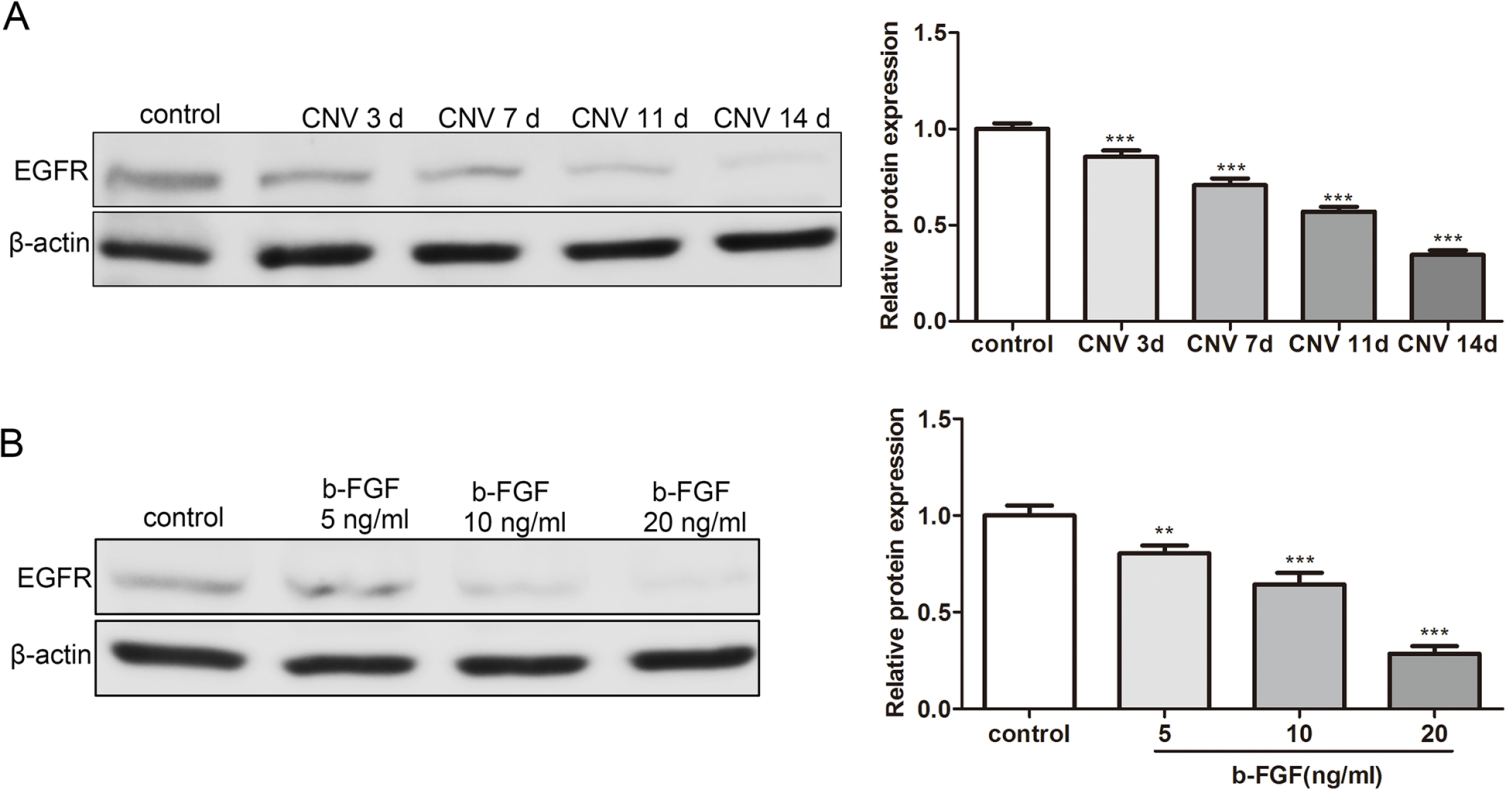
Analysis of the EGFR protein level in CRNV rats induced by corneal suture and HUVECs treated with b-FGF. **A** The CRNV rat model was established using corneal sutures. Western blot was conducted to measure the protein level of EGFR in CRNV tissues of the rats on the day 3, 7, 11 and 14 after surgery. **B** HUVECs were treated with 5, 10 or 20 ng/ml b-FGF to establish the in vitro CRNV models. The EGFR protein level was tested by Western blot. **P < 0.01 ***P < 0.001 vs. Control

Subsequently, we further evaluated the expression of EGFR in rat tissues of CRNV. As displayed in Fig. 5A, the EGFR protein level was decreased in the CRNV group, and its protein level was lowest on day 14 after surgery. Meanwhile, we assessed the EGFR protein level in HUVECs treated with b-FGF. The data corroborated that the EGFR protein level was down-regulated after the treatment of b-FGF, and the EGFR protein level was down-regulated with the increased b-FGF concentration (Fig. 5B). Taken together, these results demonstrated that the EGFR protein level was decreased in CRNV rats induced by corneal suture and HUVECs treated with b-FGF.

### Regulation of EGFR on the proliferation and migration of b-FGF-treated HUVECs

**Fig. 6:**
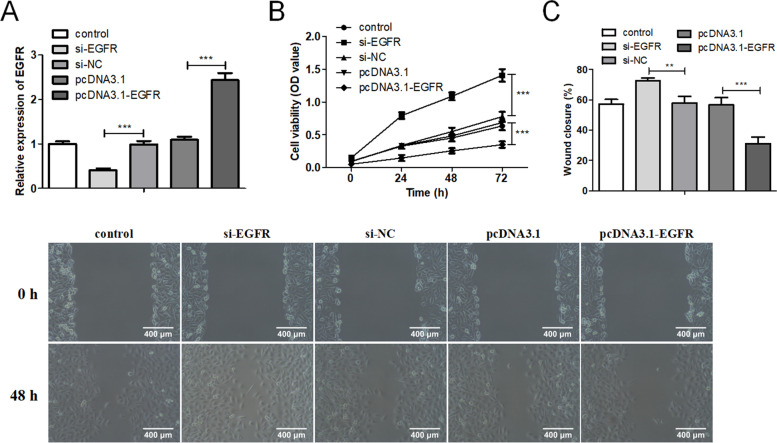
Influence of EGFR on the proliferation and migration of b-FGF-treated HUVECs. After si-EGFR or pcDNA-EGFR was transfected into HUVECs, the cells were treated with 20 ng/ml b-FGF for 24 h. **A** The transfection efficiency of si-EGFR or pcDNA-EGFR was assessed by qRT-PC. **B** Cell Counting Kit-8 (CCK-8) assay was carried out to determine the proliferation of HUVECs at 0, 24, 48 and 72 h. **C** A wound-healing assay was applied to assess the migration of HUVECs (scale bar: 400 μm). **P < 0.01 *** *P* < 0.001

Based on the finding that EGFR was lowly expressed in b-FGF-treated HUVECs, we continued to explore whether EGFR was interrelated to the regulation of the proliferation and migration of b-FGF-treated HUVECs. HUVECs were transfected with si-EGFR or pcDNA-EGFR, and the transfection efficiency was verified by qRT-PCR (Fig. 6A). CCK-8 assay indicated that the interference with EGFR promoted the proliferation of HUVECs, and the overexpression of EGFR restrained the HUVEC proliferation (Fig. 6B). Similarly, the EGFR knockdown enhanced the migration ability of HUVECs, and the EGFR overexpression showed the opposite trend (Fig. 6C). Thus, the above experimental data confirmed that the interference with EGFR facilitated the proliferation and migration of HUVECs, and the overexpression of EGFR produced the opposite trend.

### Rescue experiment verifies that miR-335 regulates the migration and angiogenesis of b-FGF-treated HUVECs via EGFR

**Fig. 7:**
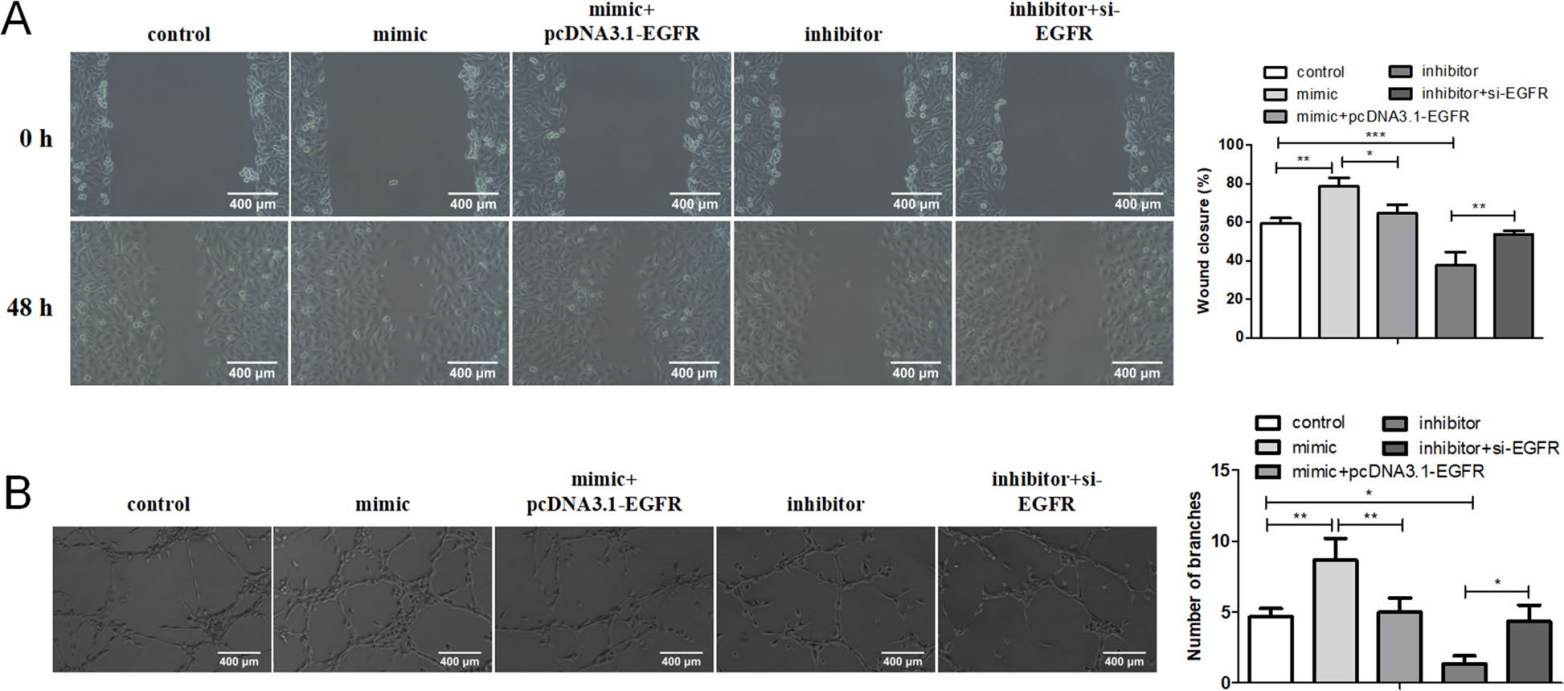
Verification of the miR-335/EGFR axis regulation on the migration and angiogenesis of b-FGF-treated HUVECs. MiR-335 mimic and pcDNA-EGFR, miR-335 inhibitor and si-EGFR were transfected into HUVECs, and the cells were then treated with 20 ng/ml b-FGF for 24 h. (**A**) The migration of HUVECs was analyzed by wound-healing assay. (**B**) Tube formation assay was conducted to assess the number of branches and the tube length (scale bar: 400 μm). *P < 0.05, ** *P* < 0.01, *** *P* < 0.001

To examine whether the miR-335/EGFR axis regulated the migration and angiogenesis of b-FGF-treated HUVECs, the miR-335 mimic and pcDNA-EGFR, miR-335 inhibitor and si-EGFR were transfected into HUVECs, and the cells were then treated with b-FGF. From the wound healing assay analysis, we found that the miR-335 overexpression promoted the migration of HUVECs, while the transfection of pcDNA-EGFR partially reversed this promotion. Meanwhile, the miR-335 knockdown repressed the HUVEC migration, and the transfection of si-EGFR partially reversed this repression (Fig. 7A). Furthermore, the transfection of miR-335 mimic increased the number of branches and the tube length, while the EGFR overexpression partially reversed this increase. The miR-335 knockdown reduced the number of branches and the tube length, while the interference with EGFR partially reversed this reduction (Fig. 7B). All in all, these data suggested that miR-335 regulated the migration and angiogenesis of b-FGF-treated HUVECs through decreasing EGFR.

## Discussion

Considering that miR-335 had the promoting function of angiogenesis [13], our current study further confirmed that miR-335 expression was up-regulated in CRNV rats induced by corneal suture and HUVECs treated with b-FGF. To our knowledge, this is the first time to investigate the expression of miR-335 in CRNV models. Based on this, our functional assays demonstrated that the interference with miR-335 repressed the migration and angiogenesis of b-FGF-treated HUVECs, which was similar to the above conclusion. To explore its mechanism, we illustrated that miR-335 targeted EGFR and miR-335 regulated the migration and angiogenesis of b-FGF-treated HUVECs through EGFR. Thus, our study might provide a novel regulatory axis for CRNV: miR-335/EGFR.

Previous studies have shown that endothelial cell function is the basis of maintaining vascular homeostasis [16, 17]. HUVECs, as one of the classical endothelial cells, have been used as an in vitro model to study angiogenesis and simulate the CRNV cell model under the stimulation of b-FGF [18]. Similarly, our study confirmed that the b-FGF treatment facilitated HUVEC migration and angiogenesis. Combined with the previous findings that miR-335 has the function of facilitating angiogenesis, this study aims to clarify whether miR-335 regulated CRNV. As expected, we found that miR-335 expression was increased in CRNV rats induced by corneal suture and HUVECs induced by b-FGF, and the miR-335 knockdown repressed the migration and angiogenesis of b-FGF-treated HUVECs, indicating that miR-335 had an essential regulatory function in the process of CRNV. Thus, we next focused on the regulatory mechanism of miR-335 in CRNV.

MiRNAs regulate cell proliferation, apoptosis and tumorigenesis through various mechanisms [19, 20]. Recently, increasing studies show that miRNAs regulate the occurrence and development of CRNV by binding to their target proteins. For instance, Kather et al. indicated that miR-204 promotes the CRNV progression by targeting angiopoietin-1 [21]; Pan et al. confirmed that miR-211 mediates the down-regulation of PROX1 protein to repress the angiogenesis of HUVECs, thus playing an essential role in the process of CRNV [22]. Importantly, we found that there were binding sites between miR-335 and EGFR through online prediction software, suggesting that miR-335 might interact with EGFR. The dual-luciferase reporter assay further verified this interaction. Meanwhile, we found that miR-335 negatively regulated the expression of EGFR.

Epidermal Growth Factor Receptor (EGFR) is a transmembrane glycoprotein and exerts critical functions in regulating various cellular responses such as cell proliferation, differentiation and survival [23, 24]. Abnormal expression of EGFR is associated with the development of CRNV. As reported, the increased EGFR expression in corneal epithelium alleviates corneal inflammation and CRNV [25]. What similar to this finding, our data illustrated that the EGFR protein level was down-regulated in CRNV rats induced by corneal suture and HUVECs treated with b-FGF. Furthermore, the overexpression of EGFR repressed the proliferation and migration of HUVECs, and the interference with EGFR had the opposite trend. Critically, our rescue experiment verified that miR-335 regulated the migration and angiogenesis of b-FGF-treated HUVECs through EGFR.

In summary, our present research verified for the first time that miR-335 was increased in CRNV rats induced by corneal suture and HUVECs induced by b-FGF. We also confirmed that miR-335 promoted the migration and angiogenesis of b-FGF-treated HUVECs by decreasing EGFR. Thus, the present study might aid in elucidating a potential therapeutic target for CRNV in the future.

## Supplementary Information

**Additional file 1.**

**Additional file 2.** 

## Data Availability

All data generated or analysed during this study are included in this published article.
