# Supplementary figures and images for: MiR-335 promotes corneal neovascularization by Targeting EGFR
Source: BMC Ophthalmol. 2022 Jun 15;22:267. doi: 10.1186/s12886-022-02481-0 (PMC9199176; doi:10.1186/s12886-022-02481-0)

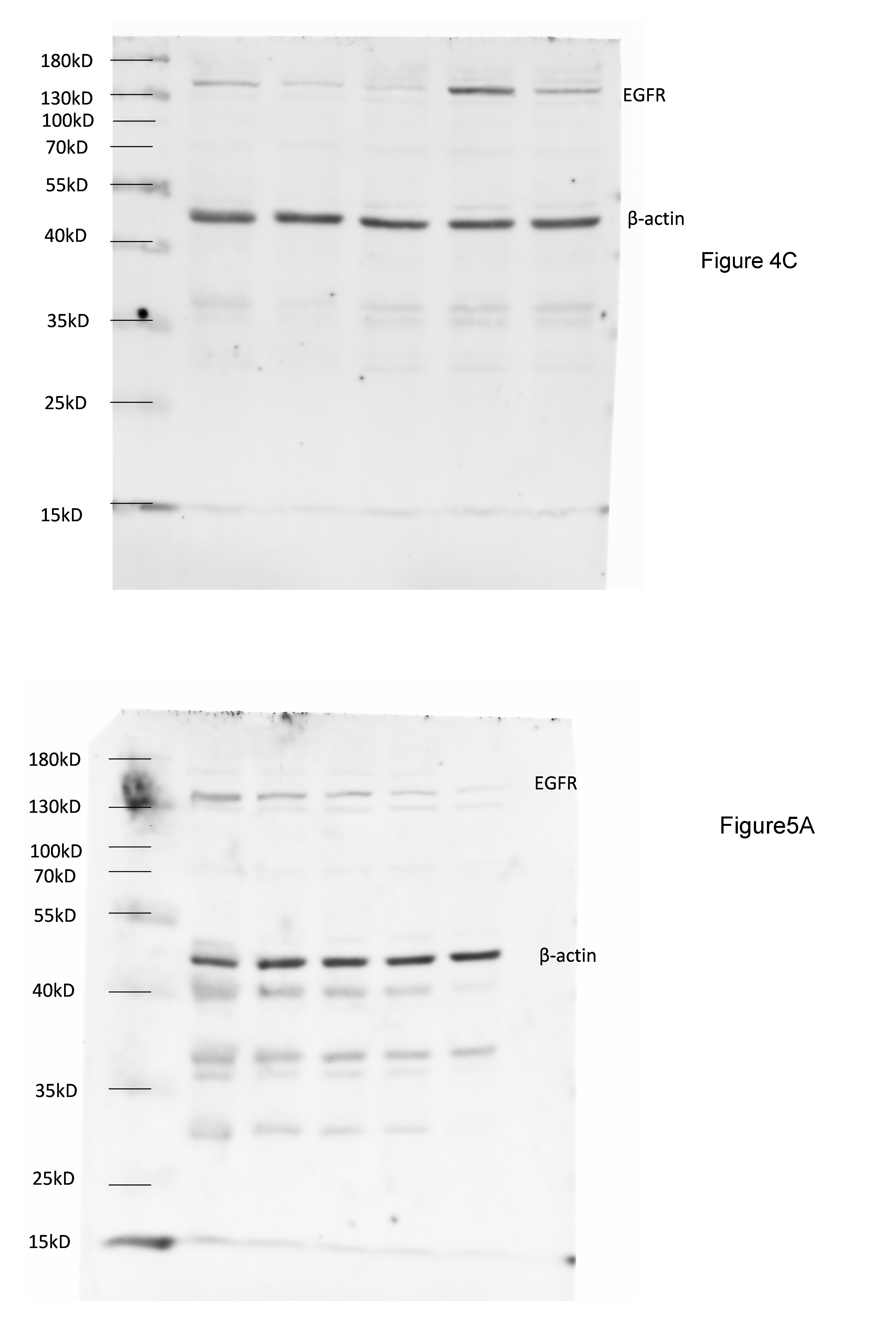

Supplement: Supplementary file 1 — Additional file 1. [file 12886_2022_2481_MOESM1_ESM.tif]

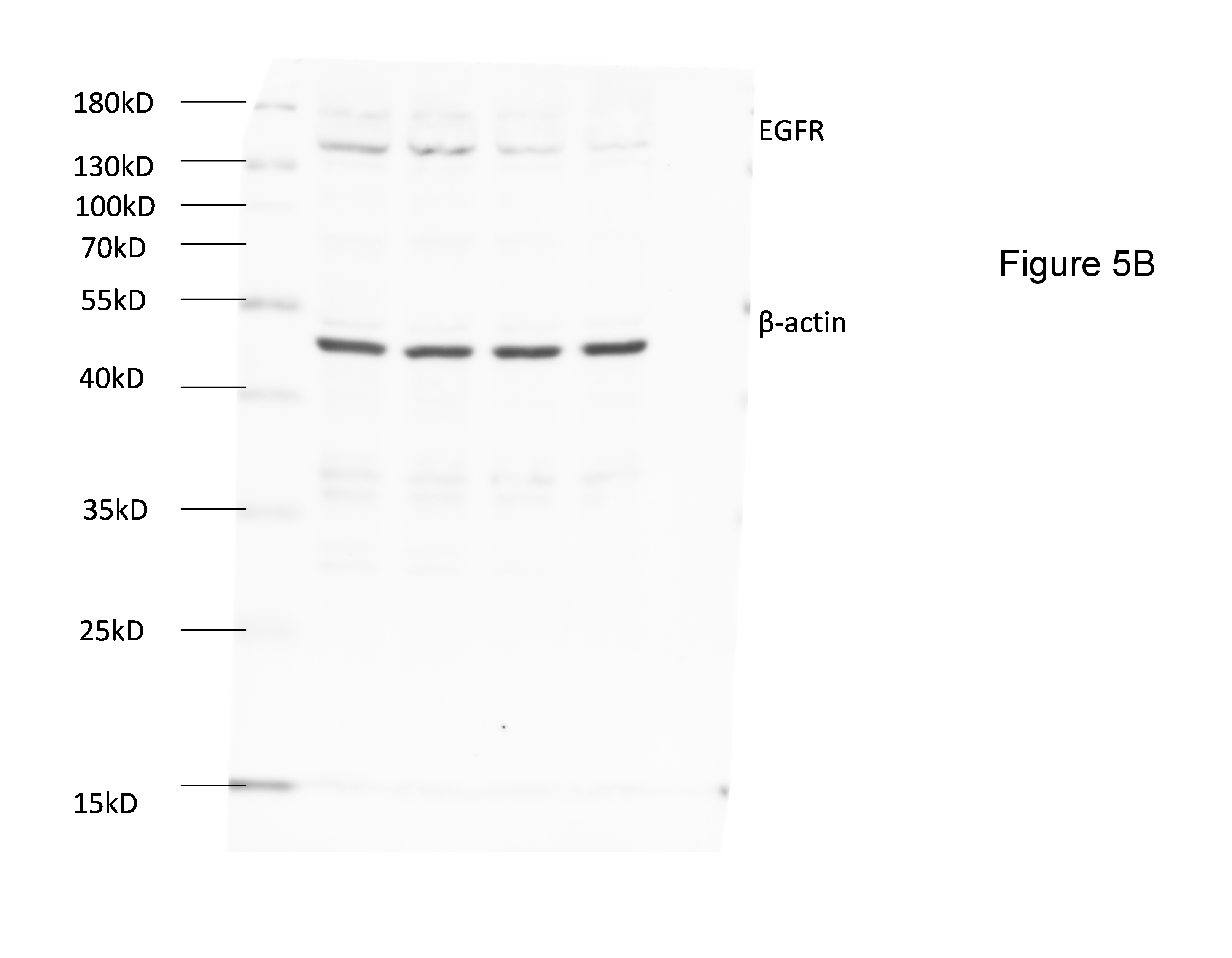

Supplement: Supplementary file 2 — Additional file 2. [file 12886_2022_2481_MOESM2_ESM.tif]
